# Cardiac surgical outcome prediction by blood pressure variability indices Poincaré plot and coefficient of variation: a retrospective study

**DOI:** 10.1186/s12871-020-00972-5

**Published:** 2020-03-03

**Authors:** Senthil Packiasabapathy, Varesh Prasad, Valluvan Rangasamy, David Popok, Xinling Xu, Victor Novack, Balachundhar Subramaniam

**Affiliations:** 1 Department of Anesthesia, Critical Care, and Pain Medicine, Beth Israel Deaconess Medical Center, Harvard Medical School, Boston, MA USA; 2 Harvard-Massachusetts Institute of Technology Program in Health Sciences and Technology, Cambridge, MA USA; 3 Institute for Medical Engineering and Science, Massachusetts Institute of Technology, Cambridge, MA USA; 4 Clinical Research Center, Soroka University Medical Center and Faculty of Health Sciences, Ben-Gurion University of the Negev, Beer-Sheva, Israel; 5 Associate Professor of Anesthesia, Harvard Medical School, Ellison “Jeep” Pierce endowed chair of Anesthesia, Director, Centre for Anesthesia Research Excellence (CARE), Beth Israel Deaconess Medical Center, One Deaconess Road, CC-650, Boston, MA 02215 USA

**Keywords:** BP variability, Poincaré plot, Coefficient of variation, Cardiac surgery, STS risk score

## Abstract

**Background:**

Recent literature suggests a significant association between blood pressure variability (BPV) and postoperative outcomes after cardiac surgery. However, its outcome prediction ability remains unclear. Current prediction models use static preoperative patient factors. We explored the ability of Poincaré plots and coefficient of variation (CV) by measuring intraoperative BPV in predicting adverse outcomes.

**Methods:**

In this retrospective, observational, cohort study, 3687 adult patients (> 18 years) undergoing cardiac surgery requiring cardio-pulmonary bypass from 2008 to 2014 were included. Blood pressure variability was computed by Poincare plots and CV. Standard descriptors (SD) SD1, SD2 were measured with Poincare plots by ellipse fitting technique. The outcomes analyzed were the 30-day mortality and postoperative renal failure. Logistic regression models adjusted for preoperative and surgical factors were constructed to evaluate the association between BPV parameters and outcomes. C-statistics were used to analyse the predictive ability.

**Results:**

Analysis found that, 99 (2.7%) patients died within 30 days and 105 (2.8%) patients suffered from in-hospital renal failure. Logistic regression models including BPV parameters (standard descriptors from Poincare plots and CV) performed poorly in predicting postoperative 30-day mortality and renal failure [Concordance(C)-Statistic around 0.5]. They did not add any significant value to the standard STS risk score [C-statistic: STS alone 0.7, STS + BPV parmeters 0.7].

**Conclusions:**

In conclusion, BP variability computed from Poincare plots and CV were not predictive of mortality and renal failure in cardiac surgical patients. Patient comorbid conditions and other preoperative factors are still the gold standard for outcome prediction. Future directions include analysis of dynamic parameters such as complexity of physiological signals in identifying high risk patients and tailoring management accordingly.

## Background

The total global surgical volume in 2012 was estimated to be 312.9 million operations per year [1]. With an increase in aging population and comorbidities, increasing number of cardiac surgeries are performed every year. Despite all the advancements in perioperative medicine, adverse outcomes still remain a concern for the cardiac surgical patient [2]. Currently risk prediction scores such as the Society of Thoracic Surgeons (STS) and European System for Cardiac Operative Risk Evaluation (EuroSCORE) most commonly use static patient comorbidities. The observed mortality was 6% compared to the predicted estimates of 19% by EUROscore and 11% by STS scoring system [3]. This demonstrates the need for better granularity in risk stratification, especially among patients with an increased risk of adverse postoperative outcomes, to aid in triaging and tailored interventions.

Advanced hemodynamic monitoring reflects the fluctuating physiological state in response to the stress of surgery and anaesthesia. Analysing these dynamic changes to infer the reserve of the patient could help to increase the specificity of the risk prediction models. Several studies showed significant association between hemodynamic derangements and major adverse events (MAE) [4–7]. However, the study by Monk et al. [7] did not find any additive value from the intraoperative hypotension compared to the preoperative variables.

Recently there has been growing interest in perioperative fluctuations in blood pressure termed as ‘blood pressure variability’ and their assocaitons with adverse outcomes [4, 8–11]. Aronson et al. [4] studied the time spent outside a specific systolic BP range in cardiac surgical patients. Mascha et al. [10] measured time weighted average and variability of intraoperative BP in noncardiac surgical patients. However, neither study described the predictive ability of the variability measures on postoperative outcome.

Poincare’ analysis, is used to measure BP variability. Poincare plot is a geometrical representation of a physiological signal and provides beat to beat information about cardiovascular system [12, 13]. It provides qualitative visualization of linear dynamic changes. It has been found as the most powerful predictor of postoperative ischemia [14] and readily detects sympathovagal changes during anaesthesia [15, 16]. In our previous work, we did a similar analysis exploring the association between BP variability measured by Coefficient of Variation (CV) and postoperative outcomes [6]. We found a significant association between CV and postoperative outcomes. In this study, we took the next step of exploring the predictive ability of blood pressure variability measured by CV and Poincare plot on postoperative outcomes. If successful, these BP variability indices by incorporating dynamic pathophysiologic characteristics could enhance the predictive ability of current risk prediction scores.

We hypothesized that Poincare plot and CV could predict postoperative outcomes better than existing risk prediction scores. In this study, we explored the ability of blood pressure variability measured by Poincaré and CV in predicting adverse outcomes among patients undergoing cardiac surgery.

## Methods

### Patient cohort

This retrospective, observational, cohort study was conducted using the data obtained from Society of Thoracic Surgery (STS) database and institutional Anesthesia Information Management Systems (AIMS) database, after the Institutional Review Board approval (IRB, Beth Israel Deaconess Medical Centre, Boston, US, Protocol #2008P000478). Informed patient consent was waived by our IRB. This manuscript adheres to the applicable Strengthening the Reporting of Observational Studies in Epidemiology (STROBE) standards for observational studies [17]. Blood pressure data were collected from a total of 3687 patients over 18 years of age who underwent cardiac surgery that required cardio-pulmonary bypass (CPB) from January 2008 to June 20,143.

### Perioperative management

Perioperative management of the patient cohort analysed in this study was along the lines of the Institute protocol during the period of data acquisition. As the type of anesthetic regimen used is an important predictor for hypotension after induction [18], we have described our anesthesia technique. In brief, anesthesia induction typically included fentanyl, Propofol or etomidate tailored to the patient profile and rocuronium for neuro-muscular blockade. Isoflurane in 100% oxygen was used for maintenance, along with supplemental boluses of fentanyl. A non-pulsatile cardiopulmonary bypass was used with the flow titrated to maintain mean arterial pressure of 50–70 mmHg and a venous oxygen saturation greater than 60%. Alpha stat pH management was employed to manage blood gases. Temperature was maintained at 34 °C in coronary artery bypass grafting (CABG) surgeries, 32 °C in valve surgeries. All patients were shifted transferred to cardiovascular intensive care unit for postoperative care.

### Data acquisition

Invasive arterial blood pressure data including systolic and mean pressures during the pre-bypass, bypass and post-bypass phases of cardiac surgery were obtained from the hospital’s anesthesia information management systems (AIMS) (CompuRecord, Philips Healthcare, Andover, MA, USA) at a rate of one sample every 15 s. Given the lack of pulsatility, systolic blood pressure (SBP) was not measured during CPB. Mean arterial pressure (MAP) was recorded during all the three phases. Patient characteristics were obtained from the STS database. This database is a clinical outcomes registry that records the care of patients undergoing cardiac procedures at participating hospitals. Patient characteristics obtained from STS include, baseline demographic data, patient characteristics such as comorbidities, medications, intraoperative characteristics, STS risk scores for morbidity and mortality, STS Predicted risk scores for renal failure, and post-operative outcomes, namely, 30-day mortality and renal failure during hospital admission.

STS risk scores were computed for each patient undergoing cardiac surgery by institutional STS coordinators as a part of nationwide STS database. Data on mortality was gathered from this STS database. If a patient was discharged and sent home, the patient was given a 30-day appointment. Those who missed the 30-day appointment were given a call by the STS database coordinator to note the morbidity and mortality. State STS coordinators also run the Social Security Death Index to capture those who died within 30 days after cardiac surgery, and this information was sent to the individual hospital.

### Data analysis

BP variability was calculated in terms of coefficient of variation (CV) and Poincaré plots. CV is defined as the standard deviation divided by mean. Poincaré plot is a quantitative, graphical tool that provides a visual representation of the non-linear aspects of a time series data sequence on a phase-space or Cartesian plane. It is a geometrical representation of a physiological signal’s time-series and provides qualitative visualization of its nonlinear dynamic changes. It is a scatter plot (AKA return map / phase delay map) where each data point on a time series (*x*_*n*_) is plotted against the next one (*x*_*n* + 1_) [13, 19]. It is a simple visual tool, the shape of which represents the variability of the time series *x*_*n*_.. The ellipse shape of the plot provides two standard descriptors SD1 and SD2 for quantifying the plot geometry [19]. The line of identity is the 45° imaginary diagonal line on the elliptical Poincaré plot. SD1 is the minor semi-axis of the fitted ellipse and measures the dispersion of data perpendicular to the line of identity. SD2 is the major semi-axis of the fitted ellipse and measures the dispersion along the line of identity. SD1 represents short-term variability, and SD2 long-term variability [19].

Poincaré plots of SBP and MAP, measured every 15 s were constructed per patient using MATLAB (Natick, MA) by producing a scatter plot of each B*P* value against the next one. SD1, SD2 were obtained from the plot using the ellipse fitting technique. This was done specifically for each phase of surgery (pre-bypass, bypass and post-bypass). BPV data was merged with patient characteristics and outcome details obtained from the Society of Thoracic Surgeons National Adult Cardiac Surgery Database (STS).

### Study outcomes

Our primary outcomes were 30-day mortality and in-hospital renal failure that were defined based on STS version 2.61 definitions for postoperative outcomes. Renal failure was defined as having one or both of: 1) increase in serum creatinine level > 2.0, and 2 x greater than baseline, 2) a new requirement for dialysis postoperatively. Mortality includes: 1) all deaths, regardless of cause, occurring during the hospitalization in which the operation was performed, even if after 30 days (including patients transferred to other acute care facilities); and (2) all deaths, regardless of cause, occurring after discharge from the hospital, but before the end of the thirtieth postoperative day. If a patient was discharged, they were given a 30-day appointment. Those who missed the 30-day appointment were contacted through phone by the STS database coordinator to note the morbidity and mortality. State STS coordinators also run the Social Security Death Index to capture those who died within 30 days after cardiac surgery, and this information was sent to the individual hospital.

### Statistical analysis

Data is presented as median (interquartile range) or n (%) depending upon the variable. Chi-square, Fischer’s exact or Mann-Whitney U test were appropriately used to assess differences in baseline characteristics, surgical and blood pressure data between groups, stratified by mortality and renal failure. Normality of continuous variables was assessed using Shapiro-Wilk test. All analyses were conducted using IBM SPSS Statistics, Version 24.0 (Armonk, NY: IBM Corp.)

A goodness of fit for a multivariable binary logistic regression model (mortality vs. no mortality, renal failure vs. no renal failure) was tested using the Hosmer-Lemeshow test. The groups and contingency table used for Hosmer-Lemeshow test were presented in Supplementary Table 1. The concordance statistic (C-statistic) was calculated to quantify the predictive strength of this ‘baseline model’ which included patient characteristics from the STS database as independent variables. The same was performed on univariable models with CV, SD1 and SD2 respectively as the predictive variables. The final models included the STS variables along with the BPV parameters to test any improvement in performance over the baseline model. In brief, the multivariable model that explored predictive ability of STS risk index alone, was adjusted to age, surgery category, STS risk score, and intraoperative vasopressor dose. In models exploring the predictive ability of BP variability indices, it was adjusted to age, surgery category, STS risk score, and intraoperative vasopressor dose. Missing STS risk algorithm scores were imputed and assessed for inclusion in the model. We considered *p* <  0.05 as statistically significant.

We included STS risk score as a variable in the models as they are used at the national level as a common metric for assessing center-to-center performance, patient counseling and clinical decision making. Moreover, it includes valuable information about patient demographics and surgical characteristics that could potentially affect the outcome after surgery and have been used as a variable in previous studies. Initial variables selection for the multivariate models were based on clinical judgement and statistical significance in univariate analysis. Further variable selection was performed in a hierarchical fashion using stepwise variable selection. Estimation was terminated at iteration number 7 because parameter estimates changed by less than .001.

### Power analysis

No a priori power or sample size calculation was performed for the study. Given the exploratory nature of the BP data analysis, all patients who met entry criteria during the study period were included in the analysis.

## Results

### Baseline characteristics

**Fig. 1:**
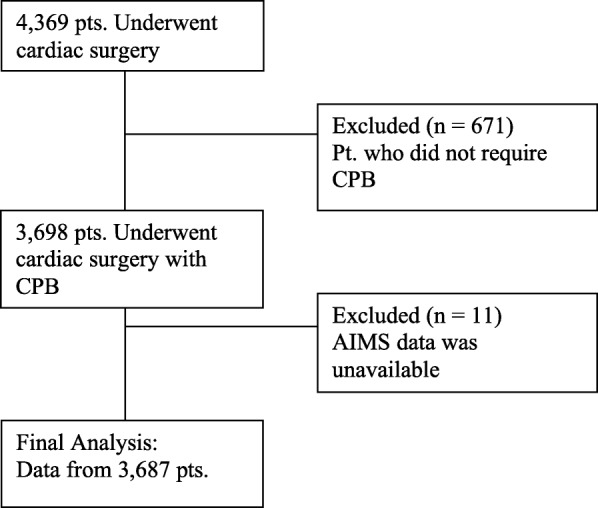
Flow chart presenting patient selection and analysis^a^. ^a^ Figure reproduced from Jinadasa SP et al. Anesth. Analg. 2018;127:832–9. Copyright© 2018 International Anesthesia Research Society

Results in this study (Fig. 1, Table 1 and Table 2) are similar to our previously published work [6] based on the same cohort of patients and copyright clearance was obtained form the publisher. 4369 patients underwent cardiac surgeries during the period of data collection (Supplementary material 1). Patietns who didn’t require CPB (*n* = 671) and those with inadequate AIMS data (*n* = 11) were excluded. A total of 3687 patients were included in the final analysis (Fig. 1). Intraoperative BP data for the whole procedure (pre-bypass, bypass, post-bypass) were not found in 309 (8.4%) patients and were excluded. CABG surgery was done in 1751 (47.5%), valve surgery in 1097 (29.8%) and combined CABG valve surgery in 725 (19.7%). From the final cohort, 99 (2.69%) died within 30 days. There was a significantly greater prevalence of congestive heart failure (*P* <  0.0001), cerebrovascular disease (*P* <  0.0001), previous myocardial infarction (*P* = 0.0002), and chronic lung disease (*P* = 0.0002) in the cohort that did not survive beyond 30 days (Table 1). They also had a significantly increased risk predicted by the STS risk prediction Algorithm for Morbidity and Mortality.

**Table 1:** Baseline Characteristics of patients stratified by mortality and renal failure^a^

Baseline Characteristics	Entire Cohort *(n = 3687)*	Survivors *(n = 3588)*	Non-Survivors *(n = 99)*	*P* Value	No Renal Failure *(n = 3582)*	Renal Failure *(n = 105)*	*P* Value
Age, *years*^b^	68 (60, 76)	68 (60, 76)	72 (62, 78)	0.002^*^	68 (60, 76)	73 (59, 81)	0.03
Male gender^c^	2565 (69.57)	2505 (69.82)	60 (60.61)	0.049^*^	2496 (69.68)	69 (65.71)	0.38
Baseline Comorbidities							
Hypertension	2900 (78.65)	2817 (78.51)	83 (83.84)	0.20	2808 (78.39)	92 (87.62)	0.02^*^
Congestive Heart Failure	1024 (27.77)	969 (27.01)	55 (55.56)	< 0.0001^*^	967 (27.00)	57 (54.29)	< 0.0001^*^
Cerebrovascular Disease	550 (14.92)	521 (14.52)	29 (29.29)	< 0.0001^*^	521 (14.54)	29 (27.62)	0.0002^*^
Dyslipidaemia	2702 (73.28)	2635 (73.44)	67 (67.68)	0.20	2631 (73.45)	71 (67.62)	0.18
Previous Myocardial Infarction	1120 (30.38)	1073 (29.91)	47 (47.47)	0.0002^*^	1082 (30.21)	38 (36.19)	0.19
Diabetes	1154 (31.30)	1116 (31.10)	38 (38.38)	0.12	1116 (31.16)	38 (36.19)	0.27
Chronic Lung Disease	499 (13.53)	473 (13.18)	26 (26.26)	0.0002^*^	476 (13.29)	23 (21.90)	0.01^*^
Dialysis	84 (6.27)	80 (2.23)	4 (4.04)	0.29	0	84	0.18
LVEF†	52.5 (50.0, 60.0)	52.5 (50.0, 60.0)	52.5 (50.0, 56.25)	0.18	52.5 (50.0, 60.0)	52.5 (50.0, 57.5)	0.17
Preoperative Medications^c^							
Aspirin	2984 (80.93)	2904 (80.94)	80 (80.81)	0.97	2900 (80.96)	84 (80.00)	0.81
β-Blockers	2714 (73.61)	2643 (73.66)	71 (71.72)	0.66	2640 (73.7)	74 (70.48)	0.46
ACE-I or ARBS	1621 (43.97)	1579 (44.01)	42 (42.42)	0.75	1575 (43.97)	46 (43.81)	0.97
Lipid Lowering	2782 (75.45)	2713 (75.61)	69 (69.70)	0.18	2706 (75.54)	76 (72.38)	0.46
Steroids	146 (3.96)	135 (3.76)	11 (11.11)	0.002^*^	139 (3.88)	7 (6.67)	0.19
Intraoperative vasopressor-inotropes. *Mg*^b^	0.63 (0.30, 1.15)	0.63 (0.30, 1.12)	0.99 (0.29, 3.03)	0.01	0.63 (0.31, 1.13)	0.69 (0.13, 2.75)	0.36
Surgical Characteristics							
Surgery Type							
CABG	1751 (47.49)	1722 (47.99)	29 (29.29)	< 0.0001^*^	1725 (48.16)	26 (24.76)	< 0.0001^*^
CABG + Valve	725 (19.66)	687 (19.15)	38 (38.38)		689 (19.24)	36 (34.29)	
Valve^d^	1097 (29.75)	1076 (29.99)	21 (21.21)		1059 (29.56)	38 (36.19)	
Other	114 (3.09)	103 (2.87)	11 (11.11)		109 (3.04)	5 (4.76)	
STS Risk Score for Morbidity and Mortality^b^	0.01 (0.01, 0.03) *n = 2732*	0.01 (0.01, 0.03) *n = 2686*	0.06 (0.02, 0.11) *n = 46*	< 0.0001^*^	0.01 (0.01, 0.03) *n = 2671*	0.05 (0.02, 0.11) *n = 61*	< 0.0001^*^
STS Predicted Risk Score for Renal Failure^b^	0.03 (0.01, 0.06) *n = 2670*	0.03 (0.01, 0.06) *n = 2625*	0.09 (0.04, 0.17) *n = 45*	< 0.0001^*^	0.03 (0.01, 0.06) *n = 2609*	0.11 (0.04, 0.21) *n = 61*	< 0.0001^*^
Bypass Period Time, *minutes*^b^							
Pre-Bypass	126.0 (104.3, 148.8)	125.8 (104.3, 148.5)	141.5 (108.3, 170.8)	0.004^*^	125.8 (104.3, 148.5)	134.8 (108.3, 161.8)	0.02^*^
Bypass	79.3 (63.8, 100.0)	79.0 (63.7, 99.0)	105.8 (75.2, 141.3)	< 0.0001^*^	79.0 (63.5, 99.0)	102.5 (75.8, 133.7)	< 0.0001^*^
Post-Bypass	76.5 (65.8, 90.8)	76.3 (65.8, 89.8)	95.5 (79.5, 135.5)	< 0.0001^*^	76.3 (65.8, 90.0)	92.3 (74.8, 120.8)	< 0.0001^*^
Cross Clamp Time, *minutes*^b^	71.0 (56.0, 91.0)	71.0 (56.0, 91.0)	93.0 (65.0, 129.0)	< 0.0001^*^	71.0 (56.0, 91.0)	94.5 (67.5, 120.0)	< 0.0001^*^

*Statistically significant at a level of significance of P <  0.05,

^a^ Figure reproduced from Jinadasa SP et al. Anesth. Analg. 2018;127:832–9. Copyright© 2018 International Anesthesia Research Society

^b^ Median [interquartile range]. ^c^ Number and %

^d^ Type of valve surgery: Aortic, Mitral, Tricuspid, Aortic + Mitral valve replacement surgeries

*ACE-I* angiotensin-converting enzyme inhibitor, *ARBs* angiotensin receptor blockers, *STS* Society of Thoracic Surgery, *CABG* coronary artery bypass graft

**Table 2:** Blood Pressure and Coefficient of Variation of patients stratified by mortality and renal failure^a^

Exposure measures	Entire Cohort *(n = 3687)*	Survivors *(n = 3588)*	Non-Survivors *(n = 99)*	*P* Value	No Renal Failure *(n = 3582)*	Renal Failure *(n = 105)*	*P* Value
Blood Pressure^b^							
*Systolic Blood Pressure*	106 (102, 111)	106 (102, 111)	102 (97, 109)	0.0002*	106 (102, 111)	103 (97, 109)	0.001*
Pre-Bypass	111 (105, 118)	111.5 (105, 118)	110 (101, 117)	0.045	111.5 (105, 118)	110 (103, 116)	0.046
Post-Bypass	100 (95, 105)	100 (95, 105)	97 (88, 103)	0.0002*	100 (95, 105)	96 (91, 104)	0.001*
*Mean Arterial Pressure*	70 (67, 73)	70 (67, 73)	66 (62, 71)	< 0.0001*	70 (67, 73)	65 (62, 69)	< 0.0001*
Pre-Bypass	78 (73, 83)	78 (73, 83)	75 (67, 80)	< 0.0001*	78 (73, 83)	75 (66, 80)	< 0.0001*
Bypass	57 (53, 62)	57 (53, 62)	58 (51, 64)	0.97	57 (53, 62)	57 (53, 63)	0.92
Post-Bypass	70 (66, 74)	70 (66, 74)	66 (62, 71)	< 0.0001*	70 (66, 74)	65 (61, 69.5)	< 0.0001*
Coefficient of Variation^b^							
*Systolic Blood Pressure*	0.21 (0.19, 0.25)	0.21 (0.19, 0.24)	0.24 (0.21, 0.27)	< 0.0001*	0.21 (0.19, 0.24)	0.23 (0.21, 0.27)	< 0.0001*
Pre-Bypass	0.20 (0.17, 0.23)	0.20 (0.17, 0.23)	0.20 (0.17, 0.25)	0.16	0.20 (0.17, 0.23)	0.21 (0.18, 0.25)	0.03*
Post-Bypass	0.18 (0.15, 0.22)	0.18 (0.15, 0.22)	0.21 (0.17, 0.24)	< 0.0001*	0.18 (0.15, 0.22)	0.20 (0.17, 0.25)	0.001*
*Mean Arterial Pressure*	0.29 (0.25, 0.37)	0.29 (0.25, 0.37)	0.31 (0.25, 0.39)	0.26	0.29 (0.25, 0.37)	0.30 (0.24, 0.38)	0.54
Pre-Bypass	0.27 (0.22, 0.38)	0.27 (0.22, 0.38)	0.27 (0.22, 0.42)	0.50	0.27 (0.22, 0.38)	0.28 (0.23, 0.40)	0.46
Bypass	0.15 (0.13, 0.18)	0.15 (0.13, 0.18)	0.15 (0.12, 0.21)	0.82	0.15 (0.13, 0.18)	0.15 (0.12, 0.20)	0.98
Post-Bypass	0.22 (0.17, 0.27)	0.22 (0.17, 0.27)	0.24 (0.19, 0.29)	0.02*	0.22 (0.17, 0.27)	0.23 (0.19, 0.28)	0.10

^a^ Figure reproduced from Jinadasa SP et al. Anesth. Analg. 2018;127:832–9. Copyright© 2018 International Anesthesia Research Society

^b^ Median [interquartile range]. *Statistically significant at a level of significance of P <  0.05

In-hospital renal failure was observed in 105 patients (2.85%). Patients who experienced renal failure had significantly greater preoperative diagnoses of hypertension (*P* = 0.02), congestive heart failure (*P* <  0.0001), cerebrovascular disease (*P* = 0.0002), and chronic lung disease (*P* = 0.01) (Table 1). They also had greater STS risk score predicted for renal failure.

### Intraoperative characteristics

Most of the patients in this cohort underwent CABG (47.49%), followed by valve surgeries, aortic surgeries etc. The overall median (IQR) duration for Pre-Bypass period was 126.0 (104.3, 148.8) minutes, Bypass 79.3 (63.8, 100.0) and Post-Bypass 76.5 (65.8, 90.8) minutes (Table 1). Significant differences were found in the median aortic cross clamp times and the duration of bypass between the cases and controls, this was significantly longer in non-survivors and in those with renal failure (Table 1). The median intraoperative SBP and MAP were significantly lower in non-survivors and in patients with renal failure. This difference in SBP and MAP was demonstrable in individual phases of surgery as well, with statistical significance (*P* <  0.05). The only exception when there was no significant difference between cases and controls was MAP during bypass (Table 2). Table 2 also depicts the median (IQR) of CV for SBP and MAP at different phases, stratified by outcome.

### Poincaré analysis

**Fig. 2:**
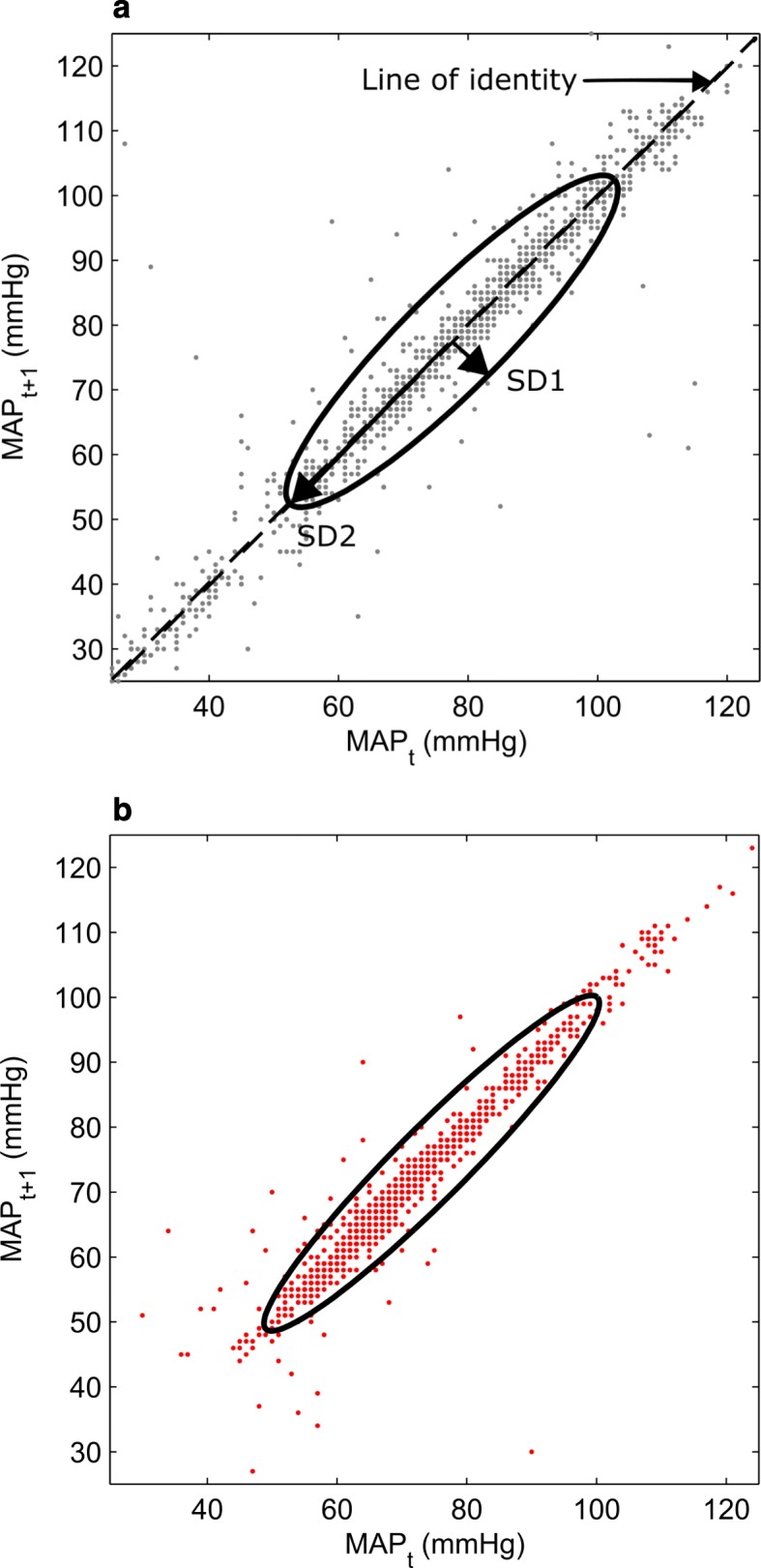
**a** Poincaré plot of a survivor, with the ellipse and derived parameters (SD1 and SD2) **b** Poincaré plot of a non-survivor, with the ellipse and derived parameters (SD1 and SD2). *MAP*_*t*_*– Mean Arterial Pressure at a data point on time series, MAP*_*t + 1*_*– Mean Arterial Pressure at a data point next on time series. SD1- minor semi-axis of the fitted ellipse, SD2- major semi-axis of the fitted ellipse*

Figure 2a presents a typical Poincaré plot of a survivor, with the ellipse and the various parameters derived out of it. Figure 2b displays the Poincaré plot of a non-survivor. The difference in shape between the plots is readily appreciable. Table 3 displays the median (IQR) of SD1, SD2 of SBP and MAP, stratified by mortality and renal failure.

**Table 3:** Poincare parameters (SD1, SD2) of SBP and MAP, stratified by mortality and renal failure

Poincare Parameters*	**Pre-bypass**			**Bypass**			**Post-bypass**		
	**No mortality (***n* = 3588)	**Mortality (***n* = 99)	***P***	**No mortality (***n* = 3588)	**Mortality (***n* = 99)	***P***	**No mortality (***n* = 3588)	**Mortality (***n* = 99)	***P***
**SBP**^**a**^									
SD1 (mmHg)	4.43 (3.32, 6.58)	4.54 (3.09, 6.53)	0.27	NA	NA	NA	3.88 (2.92, 7.52)	5.04 (3.00, 9.08)	0.04*
SD2 (mmHg)	28.46 (23.88, 34.08)	28.72 (23.51, 32.26)	0.58	NA	NA	NA	23.51 (19.77, 27.59)	25.41 (20.17, 29.94)	< 0.01*
SD1/SD2	0.15 (0.12, 0.22)	0.15 (0.11, 0.21)	0.068	NA	NA	NA	0.18 (0.13, 0.29)	0.2 (0.14, 0.35)	0.47
**MAP**^**a**^									
SD1 (mmHg)	8.11 (5.64, 10.43)	7.49 (4.77, 9.73)	0.51	2.78 (2.23, 3.48)	2.42 (1.88, 3.02)	< 0.01	8.45 (5.37, 11.15)	8.29 (6.18, 10.91)	0.25
SD2 (mmHg)	21.68 (18.24, 26.32)	21.44 (16.81, 26.89)	0.78	12.04 (9.82, 14.75)	12.13 (9.28, 14.95)	0.88	17.34 (14.24, 20.99)	16.92 (13.49, 20.20)	0.02*
SD1/SD2	0.35 (0.23, 0.47)	0.33 (0.20, 0.42)	0.25	0.24 (0.19, 0.29)	0.21 (0.16, 0.27)	< 0.01	0.46 (0.31, 0.60)	0.50 (0.37, 0.64)	0.93
	**No renal failure** (*n* = 3582)	**Renal failure** (*n* = 105)	***P***	**No renal failure** (*n* = 3582)	**Renal failure** (*n* = 105)	***P***	**No renal failure**(*n* = 3582)	**Renal failure** (*n* = 105)	***P***
**SBP**^**a**^									
SD1 (mmHg)	4.43 (3.32, 6.58)	4.36 (3.03, 6.52)	0.39	NA	NA	NA	3.88 (2.92, 7.53)	4.84 (3.00, 8.95)	0.09
SD2 (mmHg)	28.46 (23.88, 34.08)	28.72 (23.43, 35.02)	0.56	NA	NA	NA	23.50 (19.77, 27.59)	25.4 (20.35, 29.90)	0.02*
SD1/SD2	0.15 (0.12, 0.22)	0.15 (0.11, 0.21)	0.12	NA	NA	NA	0.18 (0.13, 0.29)	0.19 (0.14, 0.35)	0.23
**MAP**^**a**^									
SD1 (mmHg)	8.11 (5.64, 10.43)	7.49 (4.77, 9.73)	0.07	2.78 (2.23, 3.48)	2.44 (1.89, 3.05)	< 0.01	8.45 (5.39, 11.15)	8.29 (5.93, 10.86)	0.95
SD2 (mmHg)	21.68 (18.24, 26.32)	21.44 (16.67, 26.57)	0.53	12.04 (9.81, 14.74)	12.13 (9.37, 14.82)	0.83	17.35 (14.24, 20.99)	16.84 (13.42, 20.19)	0.16
SD1/SD2	0.35 (0.23, 0.47)	0.33 (0.19, 0.42)	0.07	0.24 (0.19, 0.29)	0.21 (0.16, 0.27)	< 0.01	0.46 (0.31, 0.60)	0.5 (0.36, 0.64)	0.06

*Statistically significant at a level of significance of P <  0.05, ^**a**^ Median [interquartile range]

*NA* Systolic Blood Pressure is not recorded during bypass due to non-pulsatile flow, *SD1* Short term variability, *SD2* Long term variability, *SBP* systolic blood pressure, *MAP* Mean arterial pressure

### Logistic regression

Goodness of fit for univariable models were performed for BPV parameters (CV, SD1, SD2) separately, corresponding to SBP and MAP specific to the phase of the surgery. These are depicted in Tables 4, 5 and 6.

**Table 4:** Univariable unadjusted models: BPV parameters

Outcome: Mortality			Outcome: Renal Failure	
Variable	C-statistic AUC (95% CI)	***P*** value	C-statistic AUC (95% CI)	***P*** value
***Systolic Blood Pressure CV***				
Pre-Bypass CV	0.541 (0.476–0.606)	0.011^*^	0.564 (0.504–0.623)	0.012^*^
Post-Bypass CV	0.621 (0.567–0.676)	< 0.001^*^	0.599 (0.537–0.660)	< 0.001^*^
***Mean Arterial Pressure CV***				
Pre-Bypass CV	0.520 (0.459–0.582)	0.127	0.522 (0.463–0.580)	0.165
Bypass CV	0.494 (0.428–0.559)	0.030^*^	0.499 (0.438–0.561)	0.085
Post-Bypass CV	0.570 (0.517–0.623)	0.123	0.548 (0.493–0.602)	0.181
***Systolic Blood Pressure SD1***				
Pre-Bypass SD1	0.536 (0.470–0.602)	0.699	0.538 (0.477–0.599)	0.445
Post-Bypass SD1	0.518 (0.458–0.578)	0.548	0.513 (0.455–0.570)	0.856
***Mean Arterial Pressure SD1***				
Pre-Bypass SD1	0.490 (0.427–0.552)	0.615	0.546 (0.484–0.608)	0.368
Bypass SD1	0.546 (0.484–0.608)	0.425	0.480 (0.419–0.541)	0.256
Post-Bypass SD1	0.539 (0.482–0.595)	0.401	0.535 (0.480–0.591)	0.088
***Systolic Blood Pressure SD2***				
Pre-Bypass SD2	0.544 (0.479 to 0.610)	0.013^*^	0.479 (0.416–0.541)	0.124
Post-Bypass SD2	0.624 (0.563–0.685)	< 0.001^*^	0.584 (0.520–0.649)	< 0.001^*^
***Mean Arterial Pressure SD2***				
Pre-Bypass SD2	0.530 (0.467–0.592)	0.015^*^	0.500 (0.436–0.564)	0.982
Bypass SD2	0.501 (0.434–0.569)	0.203	0.518 (0.455–0.580)	0.015^*^
Post-Bypass SD2	0.580 (0.524–0.637)	0.030^*^	0.491 (0.434–0.547)	0.360

*Statistically significant at a level of significance of *P* < 0.05; *BPV* blood pressure variability, *AUC* Area under the receiver operating curve, *CI* 95% Confidence Interval*, CV* Coefficient of Variation, *SD1* Short term variability, *SD2* Long term variability

**Table 5:** Predictive ability of STS risk alone for mortality and renal failure

Outcomes	C-statistic
	AUC* (CI)
**Mortality**	0.766 (0.719–0.814)
**Renal Failure**	0.734 (0.689–0.780)

*AUC Area under the receiver operating curve, *CI* 95% Confidence Interval, *STS* society of thoracic surgeons

**Table 6:** Multivariable adjusted model: BP variability parameters adjusted to age, surgery category, STS risk score, and intraoperative vasopressor dose

Outcome: Mortality			Outcome: Renal Failure	
Variable	C-statistic AUC (95% CI)	***P*** value	C-statistic AUC (95% CI)	***P*** value
***Systolic Blood Pressure CV***				
Pre-Bypass CV	0.769 (0.723–0.816)	0.109	0.739 (0.694–0.784)	0.177
Post-Bypass CV	0.780 (0.736–0.823)	0.008^*^	0.745 (0.699–0.792)	0.002^*^
***Mean Arterial Pressure CV***				
Pre-Bypass CV	0.766 (0.719–0.814)	0.870	0.734 (0.689–0.780)	0.774
Bypass CV	0.766 (0.718–0.814)	0.868	0.734 (0.688–0.780)	0.634
Post-Bypass CV	0.768 (0.721–0.815)	0.246	0.736 (0.690–0.783)	0.270
***Systolic Blood Pressure SD1***				
Pre-Bypass SD1	0.762 (0.719–0.814)	0.993	0.735 (0.689–0.781)	0.553
Post-Bypass SD1	0.767 (0.720–0.814)	0.312	0.734 (0.689–0.780)	0.498
***Mean Arterial Pressure SD1***				
Pre-Bypass SD1	0.766 (0.719–0.814)	0.946	0.731 (0.685–0.778)	0.121
Bypass SD1	0.770 (0.724–0.817)	0.238	0.732 (0.687–0.778)	0.394
Post-Bypass SD1	0.768 (0.720–0.815)	0.231	0.731 (0.684–0.779)	0.168
***Systolic Blood Pressure SD2***				
Pre-Bypass SD2	0.776 (0.732–0.820)	0.016	0.737 (0.692–0.783)	0.169
Post-Bypass SD2	0.789 (0.746–0.832)	< 0.001^*^	0.741 (0.694–0.788)	0.004^*^
***Mean Arterial Pressure SD2***				
Pre-Bypass SD2	0.768 (0.722–0.815)	0.094	0.731 (0.685–0.777)	0.405
Bypass SD2	0.765 (0.718–0.813)	0.708	0.730 (0.684–0.777)	0.067
Post-Bypass SD2	0.772 (0.725–0.819)	0.046^*^	0.731 (0.684–0.778)	0.369

*Statistically significant at a level of significance of P < 0.05, *STS* Society of Thoracic Surgeons, *AUC* Area under the receiver operating curve, *CI* 95% Confidence Interval, *CV* Coefficient of Variation, *SD1* Short term variability, *SD2* Long term variability

Results of univariable unadjusted models for BPV parameters were shown in Table 4. In general, these variables performed poorly in predicting both 30-day postoperative mortality as well as in-hospital renal failure (C-Statistic around 0.5). Statistical significance (*P* <  0.05) was observed for SBP: 1) Pre, Post-Bypass CV and SD2 for mortality, 2) Pre, Post-Bypass CV for renal failure 3) Post-Bypass SD2 for renal failure. For MAP: 1) Bypass CV for mortality 2) Pre, Post-Bypass SD2 for mortality and 3) Bypass SD2 for renal failure. Despite the above-mentioned statistical significance, the C-statistic in these cases were close to 0.5, implying a poor predictive ability.

Table 5 presents the results of the predictive ability of standard STS risk index alone for adverse outcomes. It demostrated a strong predictive power for both mortality (C-statistic: 0.766; 95% confidence interval [CI], 0.719–0.814; *P* <  0.001) and renal failure (C-statistic: 0.734; 95% CI, 0.689–0.780; *P* <  0.001). The final models were multivariable models of BPV adjusted for age, surgery category, STS risk score, and intraoperative vasopressor-inotrope dose and goodness of fit was tested for CV, SD1 and SD2 separately (Table 6). This demonstrated a good performance of the models irrespective of the BPV parameter used. The C-statistic value in almost all the models were close to the values found in the unadjusted multivariable model (0.766 for mortality and 0.734 for renal failure), implying no significant improvement in the performance of the model after inclusion of the BPV parameters.

## Discussion

In this study we used BP variability namely the Poincaré descriptors (SD1, SD2) alongwith CV. SD1, SD2 have been widely used to describe heart rate variability and we have utilized them in computing BP variability during cardiac surgeries. We found that BPV in terms of CV, SD1, SD2 did not add much value to the risk predictive performance of standard STS risk prediction index.

A number of models and scoring system for risk prediction in the context of cardiac surgery are available like the STS, EuroSCORE, NBI, CCF risk scoring system, French system etc. They have their innate limitations in that they predominantly consider static patient factors such as comorbidities, medications and nature of surgeries as the independent variables. This limitation is reflected by the fact that these models do not perform well enough towards the high-risk and elderly patient spectrum [20]. This lack of specificity was documented by the increase in gap between the predicted and observed mortality rates in high risk octogenarians [3]. In addition, the objective variables comprising varying risk indices (such as age, gender, type of surgery, coexisting illnesses such as hypertension, ejection fraction) are very crude and only apply at the population level. These models were developed to compare different institutions and providers and not meant for assigning a risk category to individual patients [21, 22].

The predictive value of these risk models was measured in terms of Shannon index and they were found to have good predictive ability for survivors, but distinctly failed to predict non-survivors [22]. Incorporating dynamic parameters in these models to improve their performance has been a subject of research in the past few years. In a recent study on BP complexity quantified by multi-scale entropy (MSE), dynamical complexity of preoperative BP was found to have an inverse correlation with risk prediction scores by the STS and EuroSCORE II indices [23].

Various tools have been used to quantify BP variability and each of them has come up with a differing magnitude and direction of association with perioperative mortality and other adverse events. Aronson et al. [4] analysed the area under the curve for SBP beyond the threshold of 95–135 mmHg, which included both the magnitude and duration of excursion beyond the thresholds. They found a positive association between the duration of excursion beyond the thresholds and increased 30-day mortality [4]. Levin and colleagues used lability, defined as the modulus of percentage change in MAP. They found an inverse association between the number of episodes of lability and the 30-day mortality [9]. Mascha et al. [10] calculated the time-weighted averages of the mean arterial pressures (TWA-MAP) and also the average real variability of the mean arterial pressure (ARV-MAP) as a measure of variability. They found a strong association of lower TWA-MAP with 30-day mortality.

In our previous analysis of intraoperative BP variability, we found a significant association between increasing systolic BPV quantified by increasing quartiles of CV and mortality and renal failure [6]. On a phase specific analysis, this association was found to be driven by CV of SBP in the post-bypass phase. However, we were not able to determine whether this association would help to prospectively identify high risk patients. In this study we observed that CV did not perform well in predictive models. The above-described analytical techniques do not describe the temporal dynamics of the BP waveform. In a feasibility study of non-linear BP dynamics, Subramaniam et al. used multi-scale entropy to assess complexity [24]. They showed that complexity of post-bypass systolic, diastolic and pulse pressures were significantly lower in non-survivors. This difference between survivors and non-survivors was not seen in standard deviation of the BP time series. This again emphasizes the superiority of dynamic over static measures.

A Poincaré plot is a quantitative, graphical tool that provides a visual representation of the non-linear aspects of a time series data sequence on a phase-space or Cartesian plane [13]. Each data point on the time-series is plotted against the subsequent data point. In a non-linear data sequence, each data point can have its influence on few or more subsequent data points. This contributes to the short-term and the long-term variability of the sequence. There are a number of descriptors being used to quantitatively describe the information conveyed by the Poincaré plot [19]. By far the most widely used technique is the ellipse fitting technique. This involves fitting an ellipse into the shape of the plot, with the center of the ellipse aligned to the center point of the plot [25]. The metrics obtained from the ellipse include the short and long semi-axes, which correspond to SD1 and SD2 respectively [25].

In our study, the predictive ability from Poincare plots were not statistically significant. One possible explanation must be the fact that Poincare plots might not describe the temporal dynamic changes in blood pressure. The limitation of these measures of BP variability like CV/Poincare is that they do not take into consideration the temporal structure of a sequence of measurements. For example, the following two sequences: A = {1 2 3 2 1 2 3 2 1 2 3 2 1} and B = {1 1 1 1 2 2 2 2 2 2 3 3 3}, have the same variability, as measured by amplitude of range and standard deviation, but completely different structures. In fact, while sequence A defines a triangular wave, sequence B is a step function [24]. One of the properties of complex waveforms includes non-stationarity [26]. Non-stationarity describes the change over time of the statistical properties of the waveform (mean, standard deviation). Though SD1 and SD2 are measures of short and long-term variabilities, they may be short handed in capturing this complex dynamic nature [13, 19]. Measures that are sensitive to the temporal changes in blood pressure might be able to predict outcomes better. It is possible that the use other measures of complexity such as the multi-scale entropy, compression and conditional entropy may significantly add to the performance of the current models.

Our study has several strengths and limitations. We analysed BP data from a large number of patients. It is also a fact that Poincaré plot has been used for the first time in cardiac surgical patients. Data involves continuous Intra-arterial blood pressure, with sampling every 15 s, which provides a very good temporal resolution, though we were not able to collect beat-to-beat pressures. We do not know if this could in any way alter the geometry of the Poincaré plot and its descriptors. Despite the large number of patients studied, data collection and analysis have been retrospective in nature, and any correlation that could be demonstrated is a mere association and a causal relationship could not be established. The descriptors SD1 and SD2 used in this study have their innate limitations in their ability to convey the non-linear, dynamic aspects of the BPV portrayed by the Poincaré plot. Finally, we didn’t explore the relationship with EuroSCORE and other risk prediction indices in this study.

## Conclusions

In conclusion, blood pressure variability computed from Poincare plots and CV were not predictive of mortality and renal failure in cardiac surgical patients. Patient comorbid conditions and other preoperative factors are still the gold standard for outcome prediction. Future holds scope for research on variables aimed at improving the discriminatory power of current risk prediction models. Our study emphasizes the need to analyse dynamic parameters such as complexity of physiological signals and explore their relationship with postoperative outcomes.

## Supplementary information

**Additional file 1.** Supplementary Table 1 Groups and contingency table for Hosmer and Lemeshow test.

**Additional file 2.** Supplementary Material 1 Dataset used in this study.

## Data Availability

All data generated or analyzed during this study are included in this published article and its supplementary information files.

## Abbreviations

AIMS: Anesthesia information management systems
ARV-MAP: Average real variability of the mean arterial pressure
BPV: Blood pressure variability
CABG: Coronary artery bypass grafting
CPB: Cardio-pulmonary bypass
C-statistics: Concordance statistics
CV: Coefficient of variation
EuroSCORE: European System for Cardiac Operative Risk Evaluation
IRB: Institutional Review Board approval
MAE: Major adverse events
MAP: Mean arterial pressure
SBP: Systolic blood pressure
SD1, SD2: Standard descriptors
STROBE: Strengthening the Reporting of Observational Studies in Epidemiology
STS: Society of Thoracic Surgeons
TWA-MAP: Time-weighted averages of the mean arterial pressures
