# Supplementary material for: Cardiac surgical outcome prediction by blood pressure variability indices Poincaré plot and coefficient of variation: a retrospective study
Source: BMC Anesthesiol. 2020 Mar 3;20:56. doi: 10.1186/s12871-020-00972-5 (PMC7055104; doi:10.1186/s12871-020-00972-5)
Supplement: Supplementary file 1 — Additional file 1. Supplementary Table 1 Groups and contingency table for Hosmer and Lemeshow test. [file 12871_2020_972_MOESM1_ESM.docx]

**Supplementary Table 1: Groups and contingency table for Hosmer and Lemeshow test**

| **Hosmer and Lemeshow Test** | | | | | | | |  |  |  |
| --- | --- | --- | --- | --- | --- | --- | --- | --- | --- | --- |
| Step | Chi-square | | | df | | Sig. | |  |  |  |
| 1 | 12.586 | | | 8 | | .127 | |  |  |  |
| **Contingency Table for Hosmer and Lemeshow Test** | | | | | | | | | | |
|  | | | Mortality = 0 | | | | Mortality = 1 | | | Total |
|  |  |  | Observed | | Expected | | Observed | | Expected |  |
| Step 1 | | 1 | 334 | | 336.166 | | 4 | | 1.834 | 338 |
|  |  | 2 | 332 | | 335.104 | | 6 | | 2.896 | 338 |
|  |  | 3 | 335 | | 334.307 | | 3 | | 3.693 | 338 |
|  |  | 4 | 333 | | 333.657 | | 5 | | 4.343 | 338 |
|  |  | 5 | 338 | | 333.002 | | 0 | | 4.998 | 338 |
|  |  | 6 | 333 | | 332.176 | | 5 | | 5.824 | 338 |
|  |  | 7 | 333 | | 330.942 | | 5 | | 7.058 | 338 |
|  |  | 8 | 330 | | 328.699 | | 8 | | 9.301 | 338 |
|  |  | 9 | 323 | | 324.660 | | 15 | | 13.340 | 338 |
|  |  | 10 | 308 | | 310.288 | | 28 | | 25.712 | 336 |
